# 

**Published:** 1882-12-20

**Authors:** 


### TABLE OF CONTENTS.

ORIGINAL AND SELECTED ARTICLES.

A Case of Severe Uterine Hemorrhage, two weeks after Delivery, by T. B. Greenley, M. D., of Kentucky, 441. Midwifery Extraordinary, by J. Hendree, M.D., of Alabama, 444. On the use of Viburnum Opulus (L.) in Dysmenorrhœa and Uterine Pain, by A. M. E. Purdy, M.D., 445. On some new Methods of Treating Hydrocele, by Robert F. Weir, M.D., 449. Surgical Clinic of Prof. W. W. Dawson, at the Good Samaritan Hospital, 453. Fatal Chloroform Narcosis Administered in the Operation of Teeth Extraction, by J. F. Byrn, M.D., of Murfreesboro, Tenn., 455. A Clinical Lecture on Dyspeptic Vertigo, by Alonzo Clark, M.D., 457. Fracture of the Odontoid Process—A Clinical Lecture, by Stephen Smith, M D., New York, 458.

ABSTRACTS AND GLEANINGS.

Skin Grafting, 461. Vesico-Vaginal Fistula Cured by Position, 462. Sensible; To keep the Hypodermic Syringe in Order, 463. Treatment of Gonorrhœa by Injections of Sulphurous Acid Diluted with water; Faith as an element of Success in Medicine, 464. The Antiseptic Treatment of Typhoid Fever, 465. Malaria in Skin Diseases—A Correction, 466. Puncture and Aspiration in Intestinal Invagination; Curing Disease by Anointing with Oil, 467. Mustard in the Treatment of Small-Pox; Treatment of Cerebro-Spinal Meningitis, 468. How Parturition is managed by the Beniamir Arabs and the Abyssinians; New Method of Reducing in Dislocation of the Humerus; Eczematous Ulcer of the Leg, 469. Abdominal Deliveries in the United States for 1880; Chloroforming Mice; Transplantation of Muscle from the Dog to Man; Extraction of Teeth During Pregnancy, 470.

SCIENTIFIC ITEMS.

Gun-Powder as a Motive Power; A Series of Magnetic Observations; The Tails of Comets; The new Metal, 471. The Microscopists at Dinner; Chemical Deep-Sea Soundings, 472.

PRACTICAL NOTES AND FORMULÆ.

Diarrhœa Pills; Soap Liniment; Algerian Corn Cure, 473. A Stimulating Expectorant; Pills for Constipation; Tape Worm; The Aromatic Powder of Chalk and Opium; Naquet's Hair-Dye, Modified, 474.

EDITORIALS AND MISCELLANEOUS.

Greetings; The Ins and the Outs, 475. Surgery in the New College; The New Pharmacopœia; Abstracts; Mortality of Whites and Blacks in the U. S. Army, 476. Medical Literature in the South; Typho-Malarial Fever, 477. New Hampshire Medical Society, 478. Inverted Toe-Nails; Doctors in Canada; Book Notices, 479. Special Notices, 480

